# The Influence of Precipitate Morphology on the Growth of Austenite Grain in Nb-Ti-Al Microalloyed Steels

**DOI:** 10.3390/ma15093176

**Published:** 2022-04-27

**Authors:** Junfeng Yuan, Yang Xiao, Na Min, Wei Li, Sixin Zhao

**Affiliations:** 1 School of Materials Science and Engineering, Shanghai University, Shanghai 200444, China; yjf970611@shu.edu.cn (J.Y.); xiaoyang7661@shu.edu.cn (Y.X.); 2 Laboratory for Microstructures, Shanghai University, Shanghai 200444, China; 3 Shanghai Key Laboratory of Materials Laser Processing and Modification, Shanghai Jiao Tong University, Shanghai 200240, China; weilee@sjtu.edu.cn; 4 School of Materials Science and Engineering, Shanghai Jiao Tong University, Shanghai 200240, China; 5 Long Products Research Institute, R&D Center, Baoshan Iron & Steel Co., Ltd., Shanghai 201900, China; zhaosixin@baosteel.com

**Keywords:** microalloyed steels, precipitate, austenite grain growth, pinning pressure

## Abstract

The present study investigates the morphological evolution of carbonitrides and the effect of these precipitates on grain boundary pinning during pseudo-carburizing a Nb-Ti-Al microalloyed steel. The result indicated that three kinds of complex precipitates with different morphologies containing Nb, Ti, and Al respectively were observed in samples austenitized at different temperatures and times. The NbC and TiN precipitates played an important role in pinning grain boundaries and suppressing the growth of austenite grains, relying on the high thermal stability of TiN precipitates and small size of NbC precipitates. The precipitate characteristics affected the size of austenite grain. Based on the Zener pinning model, the effect of precipitate characteristic on austenite grain size was quantitatively analyzed. It is found that the existence of NbC and TiN precipitated at high temperature makes austenite grain growth difficult when austenite grain boundaries were pinned by fine and diffused precipitates.

## 1. Introduction

In order to improve the wear resistance and fatigue strength, the surface of gears typically needs to be carburized. In carburizing processing, the carbon diffusion into the steels is usually conducted at temperatures between 870 and 980 °C for times from a few hours to a day [1]. Increasing the carburization temperature can reduce the processing time [1], thereby improving the productivity and saving manufacturing cost. According to Harris equation, in order to obtain a carburization depth of 1 mm, 12 h is required at a carburization temperature of 930 °C, while it is only 3 h at 1050 °C [2]. However, with the increasing of carburizing temperature, austenite grain coarsens, which affects the mechanical properties of gear steels such as fatigue strength and impact resistance [3]. Austenite grain size has an important effect on the mechanical properties of steels. The hardness, yield strength, and tensile strength increase with decreasing of austenite grain size at room temperature [4].

Several studies have indicated alloy elements and process modifications to produce suitable precipitate distributions to control austenite grain coarsening at different carburizing temperatures [5,6]. Microalloying elements, such as Nb, Ti, V, and Al, form precipitates enhancing the mechanical properties of steels by pinning austenite grain boundaries to control austenite grain growth [7,8,9,10,11,12,13,14,15] or by strengthening the matrix [1,2,3,4,5]. Much work has been carried out on austenite grain control by TiN particles, since TiN nitrides have excellent thermal stability [16,17]. At the austenitizing temperature of above 1200 °C, TiN precipitates existed abundantly in the matrix and showed dispersed distribution [18]. Previous studies have found that the size of TiN ranged from 40 to 200 nm at 1050 °C [19] and 150 to 240 nm at 1350 °C [20]. Nevertheless, when the size of carbonitrides is greater than 100 nm, the pinning effect of austenite grain boundaries weakened [21]. Sha et al. [20] present that Ti-rich nitrides remained effective for grain growth inhabitation of coarse-grained austenite at temperature below 1250 °C, despite the growth of Ti-rich nitrides at elevated equalization temperatures. One effective method to optimize the pinning effect of precipitate is considering multiple microalloyed elements such as Al, Nb, and Ti. Graux et al. [22] present that TiN precipitates were found after 30 min at 1280 °C in Ti-Nb microalloyed steel. However, after 240 min at 1050 °C, and 30 min at 1200 °C, (Ti,Nb)C precipitates can be found in the heat-treated samples, and the mean radius of (Ti,Nb)C was 66 nm and 67 nm respectively. Genki et al. [17] present that fine AlN-NbC combined particles are mainly formed in a SCM420-based case-hardening steel containing 0.035 wt.% Al and 0.032 wt% Nb, which can suppress the abnormal grain growth.

On the other hand, some authors considered that the steels with mixed additions of titanium, vanadium, and niobium show some inconsistencies in grain coarsening behavior [8,23]. The addition of niobium or vanadium to microalloyed titanium gear steel results in the formation of complex titanium–niobium or titanium–vanadium carbonitrides, since Nb, Ti, and V carbonitrides extend mutual solubilities which result in the complex carbonitrides [5]. The coarsening of precipitate particles during austenitizing result in the weakening of pinning pressure [24]. Precipitates lose the ability of suppressing austenite grain coarsening. While the previous studies have illustrated the potential of utilizing complex precipitates to limit austenite grain coarsening during carburizing, there have been no systematic studies to provide the information to optimize morphological characteristics and distributions of the precipitates to minimize grain coarsening during carburizing. Thus, the austenite grain growth in presence of multiple microalloying elements is an area that deserves further attention.

The main aim of this work is to study the influence of morphology and distributions of complex precipitate to minimize grain coarsening during pseudo-carburizing. The pinning of the fine precipitates on the austenite grain boundaries is clarified by analyzing the dissolution and coarsening of the precipitates and the pinning pressure of fine precipitates.

## 2. Experimental Procedure

**Figure 1:**
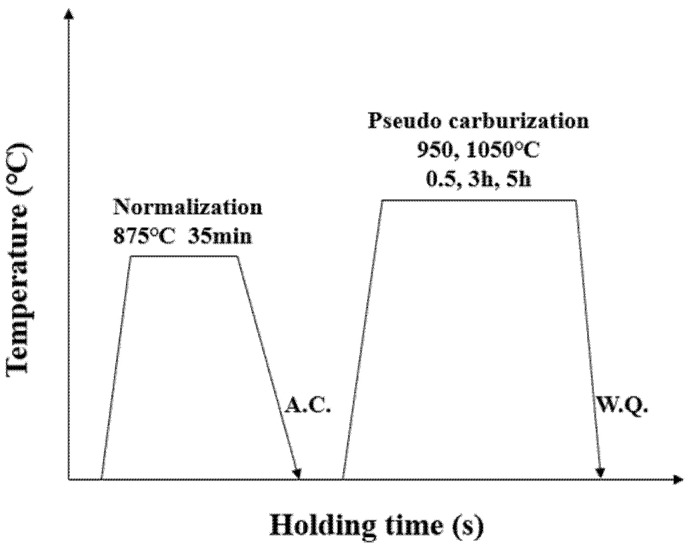
Schematic illustration of experimental process for pseudo-carburizing. A.C. denotes air cooling, and W.Q. denotes water quenching.

The 20CrMn steel is a commonly used gear steel according to China National Standard GB/T 3077-1988. The experimental steel investigated in this study is a commercial 20CrMn steel adding microalloying elements such as Nb, Ti, and Al, produced by electrical furnace melting. The main alloying elements of the investigated steels are listed in Table 1. The SPECTRO MAXx (LMX16) direct-reading spectrometer was used to determine chemical composition in this study. Figure 1 shows the heat treatment processes given to the steels. The as-received steel was normalized at 875 °C for 35 min, followed by air cooling. To investigate the effect of austenitizing temperature and holding time on the austenite grain sizes, 10 × 10 × 15 mm^3^ cube specimens were machined from the normalized initial steel, and heated to different austenitizing temperatures at 950 °C and 1050 °C for 0.5 h, 3 h, and 5 h, followed by water quenching (pseudo carburization).

To reveal the prior austenite grain boundaries, the specimens were polished by conventional metallographic techniques and etched using a saturated picric acid aqueous solution at 60 °C for 5 min. The microstructure was characterized by means of a Nikon EPIPHOT 300 optical microscopy (OM). The prior austenite grain sizes were measured with linear intercept method in accordance with GB\T 6394-2017, ensuring that at least 300 grain intersections were counted for each of the samples.

The precipitation particles were observed by a JEOL 2100F transmission electron microscope (TEM). TEM observation was operated at 200 kV on carbon replicas. Carbon extraction replicas were prepared from selected heat-treated samples. After polishing and nital (4%) etching, a carbon layer was deposited on the etched sample using a Leica-EM-ACE600 carbon evaporator. Squares of approximately 2 × 2 mm^2^ were drawn on the carbon-coated surface using a cutter blade. Samples were placed in a 10% nital solution until the sliced carbon films start delaminating from the sample surface. A carbon layer containing the precipitates was obtained. Afterwards, the carbon replicas were rinsed in three successive ethanol baths, and placed on 400-mesh copper grids. The size of precipitation particles was examined by the Image analyzing software (Image Pro Plus 6.0, Media Cybernetics, MD, USA).

## 3. Results and Discussion

### 3.1. Evolution of the Austenite Grain Size against Temperature and Times

**Figure 2:**
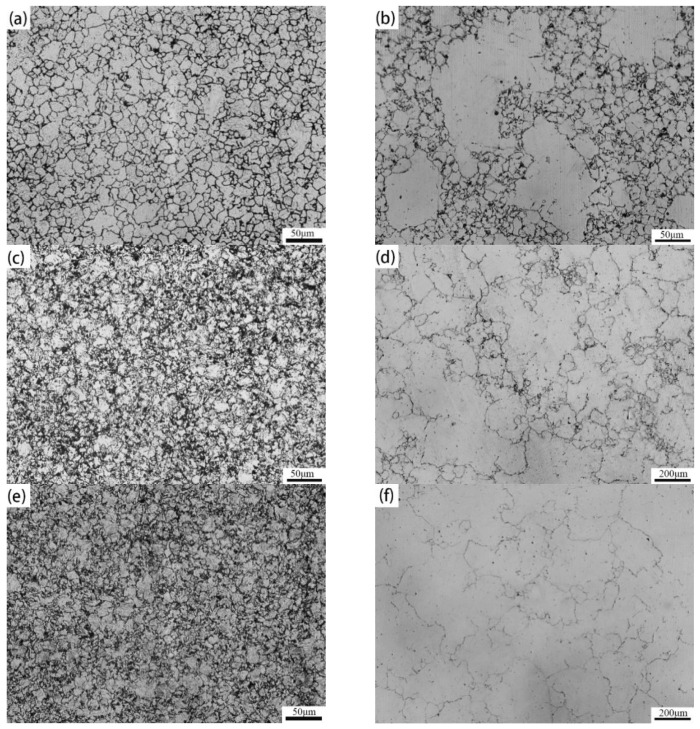
Examples of optical micrographs showing austenite grain structures after pseudo-carburizing at different temperatures for 0.5 h, 3 h, and 5 h; (**a**) 950 °C for 0.5 h; (**b**) 1050 °C for 0.5 h; (**c**) 950 °C for 3 h; (**d**) 1050 °C for 3 h; (**e**) 950 °C for 5 h; (**f**) 1050 °C for 5 h.

**Figure 3:**
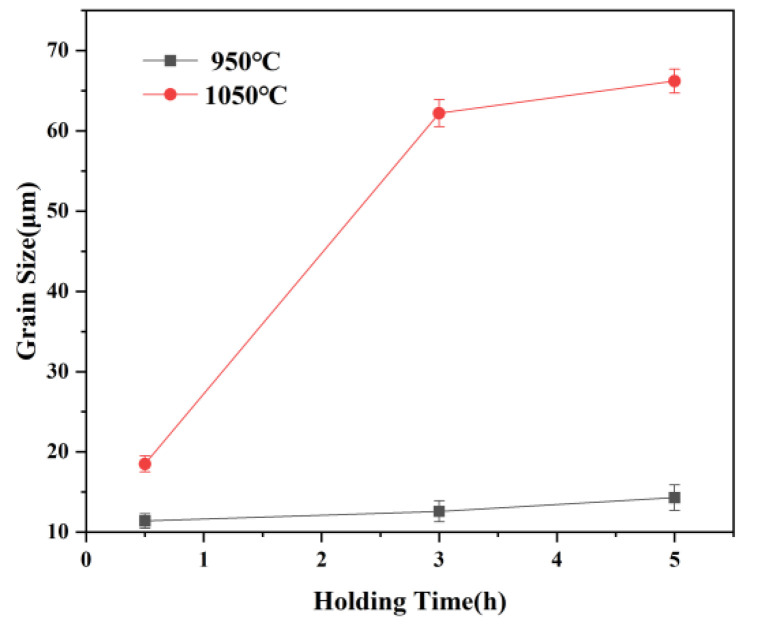
Experimental data for austenite grain coarsening against holding time at 950 °C and 1050 °C.

Figure 2 presents optical micrographs of austenite grain structures after pseudo-carburizing at 950 °C and 1050 °C for 0.5 h, 3 h, and 5 h. Figure 3 shows the evolution of the austenite grain size versus times at 950 °C and 1050 °C. As the holding time increases from 0.5 h to 5 h, the austenite grain sizes are determined as 11.4, 12.6, 14.3 μm at 950 °C and 18.5, 62.2, 66.2 μm at 1050 °C, respectively. As expected, increasing temperature and extending holding time results in growth of austenite grain. However, the growth of austenite grain does not show a law of continuous growth. At 950 °C, the austenite grain size showed a slight increase, with values between 10 and 15 μm. When the temperature rises to 1050 °C, part of austenite grains coarsened rapidly after holding for 0.5 h and duplex grain phenomenon appears obviously, as clearly shown in Figure 2b. With the extension of holding time from 3 h to 5 h, fine austenite grains coarsen as well and average austenite grain size gradually remains stable, which can be found in Figure 2d–f. The growth trend of austenite grain size is shown in detail in Figure 3. At 950 °C, extending holding time from 0.5 h to 5 h has little effect on the growth of austenite grain size. However, enhancing temperature to 1050 °C, the average austenite grain size increased obviously and subsequently remained stable with the extension of holding time, which can be speculated as the coarsening and dissolution of partial precipitates at high temperature resulting in the growth of austenite grains.

### 3.2. The Morphological Evolution of Precipitates

**Figure 4:**
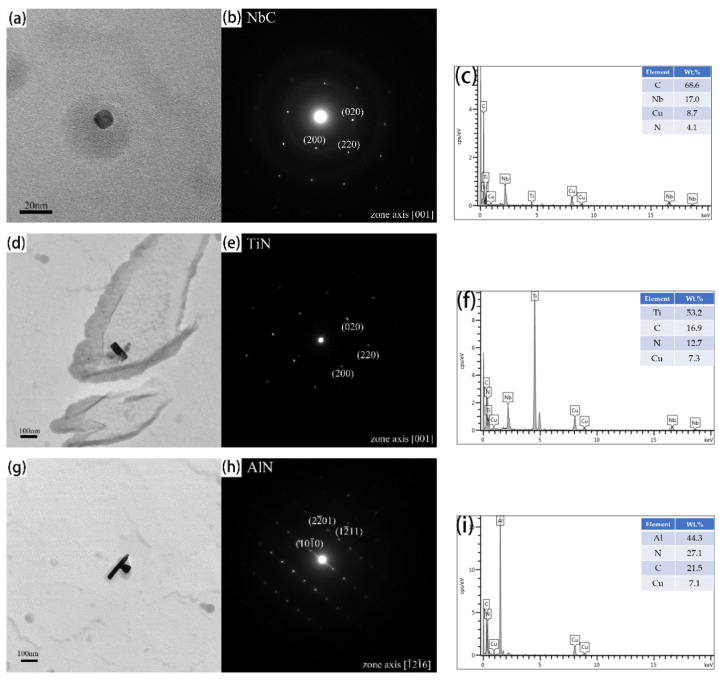
TEM micrographs of three types of precipitates found in the carbon extraction replicas, associated with selected area diffraction patterns and EDS analysis. (**a**–**c**) NbC, (**d**–**f**) TiN, (**g**–**i**) AlN.

**Figure 5:**
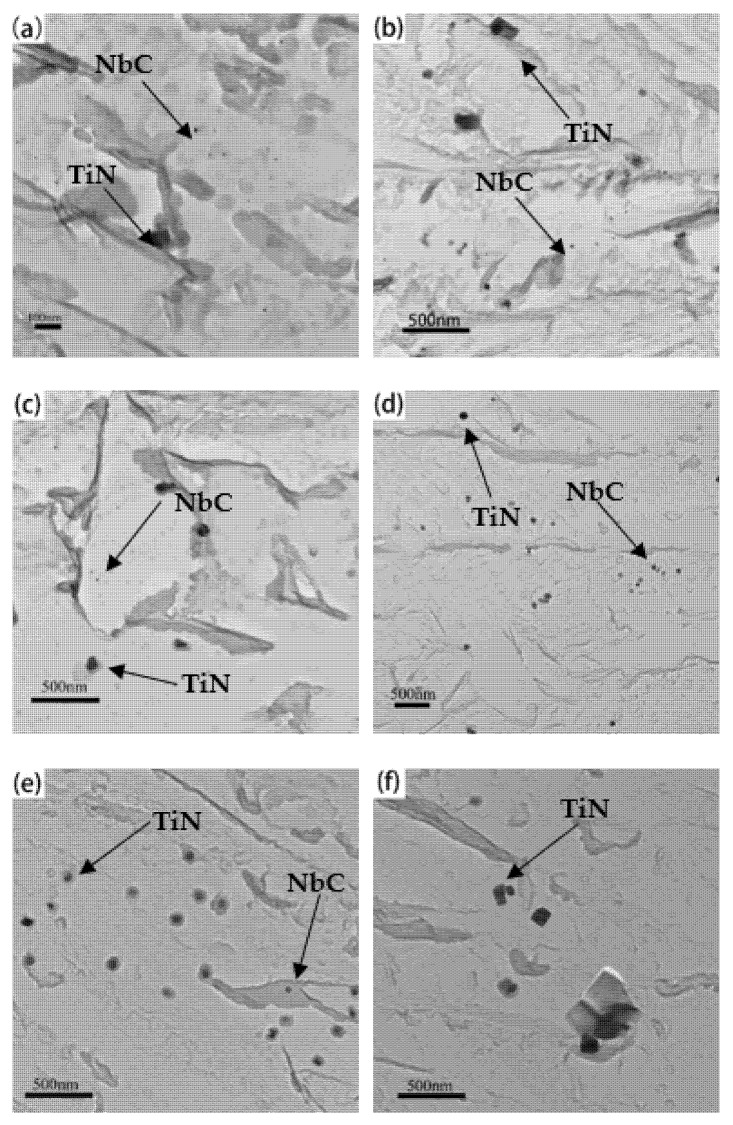
Precipitates in the carbon extraction replicas after holding at 950 °C and 1050 °C for (**a**,**b**) 0.5 h, (**c**,**d**) 3 h, (**e**,**f**) 5 h.

Figure 4 and Figure 5 show the precipitate morphologies at different heat treatments are observed by TEM. Three kinds of precipitates are identified from selected area diffraction patterns (SAED) and the energy dispersive spectroscopy (EDS) analysis (Figure 4). The differences in the size and shape of the precipitates can be clearly shown. The small precipitates are spherical (Figure 4a), and the large precipitates are cuboidal (Figure 4d). Meanwhile, a small number of rodlike precipitates can be found at austenitizing temperature of 950 °C (Figure 4g). EDS analysis showed that the spherical precipitates are rich in C and Nb elements (Figure 4c). The diffraction pattern of the spherical precipitates are confirmed with an fcc structure of space group Fm-3m, NbC. From the SAED pattern and EDS analysis (Figure 4d), the cuboidal precipitates are confirmed to be an fcc structure of space group Fm-3m, TiN with the size larger than 80 nm. By TEM and EDS analysis, a rodlike precipitate whose size is larger than 200 nm, and containing Al and N elements is confirmed (Figure 4g). The patterns can be indexed with a hexagonal structure of space group P63mc. The composition is compatible with AlN.

Figure 5 shows the type and distribution of the precipitates in the carbon extraction replicas, which clearly displays the evolution of precipitates under different austenitizing isothermal treatments. A large number of dispersed precipitates can be observed after austenitizing isothermal treatments at 950 °C and 1050 °C and the precipitates coarsen gradually with the holding time ranging from 0.5 h to 5 h. It is noteworthy that a quantity of spherical precipitates (NbC) and several dispersive distributed cuboidal precipitates (TiN) can be clearly observed in TEM micrographs, but it is difficult to capture the rodlike precipitates (AlN) in the TEM field of view, only few of them were captured during the TEM observation, as shown in Figure 4g. Ti is a strong nitride-forming element, which is easier to combine with N to form TiN, compared to Al [25]. In this study, the element addition amounts of Al, Ti, and N are 0.036%, 0.030%, and 0.0134% (wt.%), respectively. The determination error of chemical elements is 0.001 wt.%. There is not enough N to combine with Al, generating AlN precipitates during austenitizing, which results in a little amount of AlN precipitates. Therefore, the statistics of precipitate evolution only concentrate on NbC and TiN in this study.

In order to quantify the evolution of precipitates, it is necessary to get the size of precipitates and the variety of distribution. The size of spherical NbC precipitates is obtained by measuring its diameter. The size of cuboidal TiN precipitates is measured by converting to a hypothetical sphere of radius r by the following equation [26]:

(1) $$d=\sqrt{\frac{L_{A}L_{B}}{\pi }}$$

where $L_{A}$ and $L_{B}$ are the measured edge sizes of TiN precipitates.

**Figure 6:**
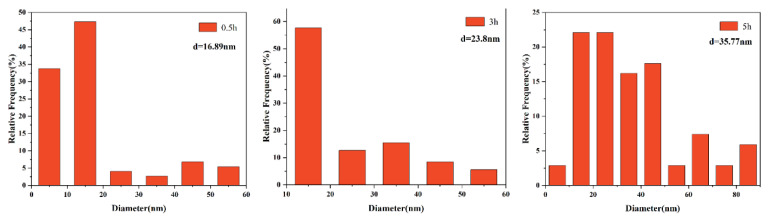
The distribution of NbC precipitates size after different holding time at the austenitizing temperature of 950 °C.

**Figure 7:**
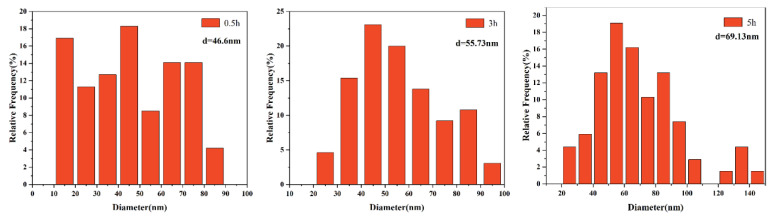
The distribution of TiN precipitates size after different holding time at the austenitizing temperature of 950 °C.

**Figure 8:**
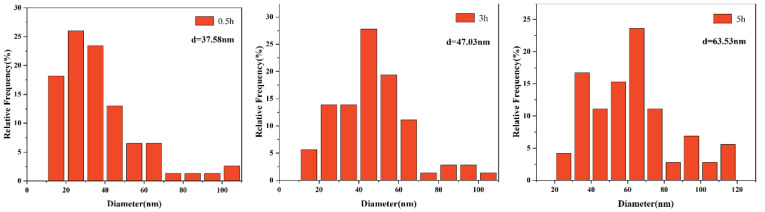
The distribution of NbC precipitates size after different holding time at the austenitizing temperature of 1050 °C.

**Figure 9:**
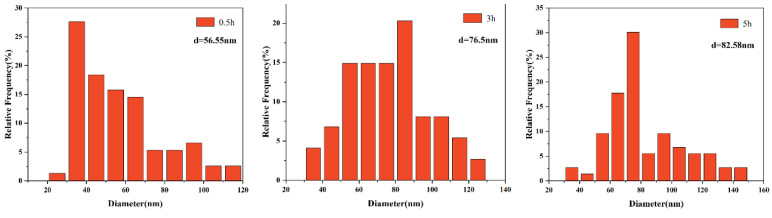
The distribution of TiN precipitates size after different holding time at the austenitizing temperature of 1050 °C.

**Figure 10:**
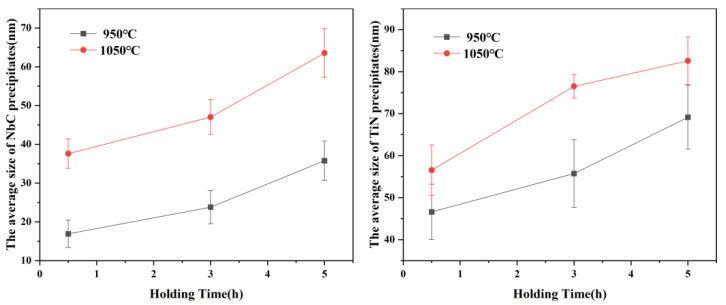
The average size of the precipitates at different temperatures with the extension of holding time.

The size distribution of precipitates is displayed in Figure 6, Figure 7, Figure 8 and Figure 9. The average size d was acquired by counting more than 200 precipitate particles. With the extension of holding time, the precipitates gradually coarsen. Figure 10 displays the curves of precipitates coarsening with the extension of holding time at 950 °C and 1050 °C. It is found that the size of both NbC and TiN precipitates increases obviously with the austenitizing temperature increase from 950 °C to 1050 °C. However, when extending the holding time at the same temperature, the size of precipitates has a much slighter increasing trend. Obviously, austenitizing temperature plays a more important role than holding time in the coarsening of precipitates.

**Figure 11:**
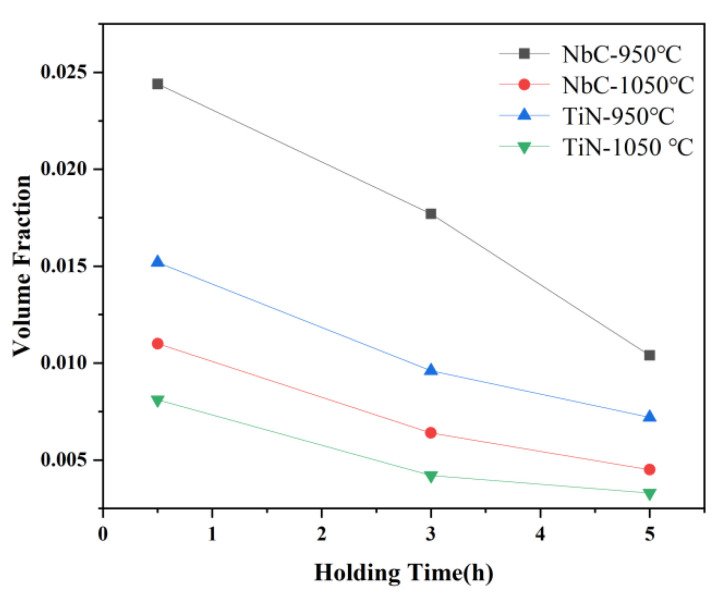
The volume fractions of the two types of precipitates under different austenitizing isothermal treatments.

In addition, coarsening and dissolution of precipitates also greatly influence the suppression of austenite grain size growth. The volume fraction of precipitates can be calculated from the results of Equation (2) [27], which reflects the amount of precipitates under different heat treatment conditions, and the results are shown in Figure 11.

(2) $$f=\frac{N\frac{4\pi }{3}r^{3}}{SD}$$

where f is the volume fraction of precipitates, N is the number of precipitates in each area, r is the precipitates radius, S is the estimated specific area, and D is the equivalent diameter of precipitates.

The volume fraction of NbC precipitates is always greater than that of TiN precipitates, whether at 950 °C or 1050 °C. When the temperature increases to 1050 °C, the volume fraction of both NbC and TiN has a significant decrease. On the other hand, the extension of holding time has a great effect on the volume fraction as well at same isothermal temperature. This phenomenon is most obvious in NbC precipitates at the isothermal temperature of 950 °C. The NbC precipitates were the most frequently observed in each sample, and has a smaller size than TiN. Although all the precipitates began to coarsen or dissolve with the increase of temperature and time, the final size of NbC precipitates was relatively small and the amount of them was relatively large. Therefore, they are likely to be the pinning particles which suppress the growth of austenite grains during the austenitizing process.

### 3.3. Effects of Precipitates on the Growth of Austenite Grain

Previous studies usually focus on the effect of temperature on precipitates evolution. In this work, different temperature and holding time are considered simultaneously to study the effect of precipitates evolution on austenite grain size. Generally, both increasing austenitizing temperature and enhancing holding time will lead to the growth of austenite grain. At the same time, the existence of precipitates can suppress the growth of austenite grain by pinning grain boundaries. It has been shown in Section 3.1 that austenite grain size of samples in this work is not significantly coarsened at 950 °C for 0.5 h, 3 h, and 5 h. However, the coarsening of austenite grain happened at 1050 °C. As holding time enhances, obvious duplex grains appear at 0.5 h, and most of the austenite grains have coarsened at 3 h. Afterwards, the grain size remain stable at holding time of 5 h. The austenite grains exhibit a nonsignificant growth at 950 °C, and obviously coarsen at higher temperature for a short time. When continue extending the holding time, the growth of grain size stopped again.

This nonlinear growth indicates that austenite grain growth is impeded, and the impediment keeps changing with the austenitizing process rather than constant. Zener et al. proposed that the precipitates on austenite grain boundaries wound impede the austenite grain growth by a pinning pressure [26]. This may explain the hindered austenite grain growth in this study. Grain growth is slowed down by precipitates generated around grain boundaries.

Equation (3) was proposed to calculate the effect of precipitates coarsening, dissolution, and distribution randomly on austenite grain growth during heat treating [28]:

(3) $$F_{z}=\beta \frac{\gamma \cdot f}{r}$$

where f is the precipitate volume fraction, r is the precipitate average radius, β is a dimensionless constant assumed to be 12, and γ is the austenite interfacial energy.

When the carbon content is lower than 0.8% (wt.%), γ can be calculated by the following equation [29]:

(4) $$\gamma =\left(0.8-0.35C^{0.68}\right)$$

Calculated by Equation (4), the interfacial energy in this study is 0.67 J·m^−2^. Due to the pinning of multiple precipitates, the total pinning pressure can be calculated by the following equation [30]:

(5) $$F_{p}=\beta \cdot \sum^{\;}\frac{\gamma \cdot f_{i}}{r_{i}}$$

where i represents all precipitate particles, in this study, including NbC, TiN, and AlN precipitates.

**Figure 12:**
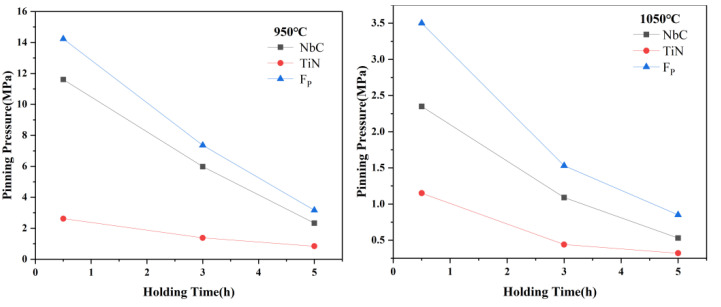
Pinning pressure of precipitates at different isothermal austenitizing treatments.

Substituting the average radius and volume fraction of precipitates calculated in Section 3.2 into Equations (3) and (5), the pinning pressure at different isothermal austenitizing treatments is obtained, as shown in Figure 12. As the extension of holding time, the pinning pressure decreases at two austenitizing temperatures. According to Equation (5), both decrease of volume fraction and increase of average radius will lead to the decrease of pinning pressure. Figure 11 shows that the volume fraction of NbC precipitates decreases rapidly with the extension of holding time at 950 °C, which results in a great decreasing trend of pinning pressure at this temperature, from 11.61 MPa in 0.5 h to 2.33 MPa in 5 h. Comparing the pinning pressure of two precipitates, the pinning pressure of NbC is always stronger than that of TiN in each isothermal austenitizing treatment, but the weakening trend of pinning pressure of NbC is larger. On the contrary, the pinning pressure of TiN maintains a relatively stable pinning pressure, from 2.62 MPa to 0.84 MPa at 950 °C, 1.15 MPa to 0.32 MPa at 1050 °C. This difference can be attributed to the lower thermal stability of NbC precipitates, which results in the serious precipitate dissolution. TiN precipitates have a great thermal stability. Although the amount of TiN precipitates is small, they are not completely dissolved.

In general, two precipitates work together to pin the austenite grain boundaries. The pinning pressure of different precipitates depends on their thermal stability and particle size. NbC maintains a fine precipitates size and TiN has better thermal stability. Figure 3 shows the austenite grain growth rate rapidly increases when temperature increases from 950 °C to 1050 °C, especially from 0.5 h to 3 h. This suggests that the microstructure evolution (volume fraction and size) has a significant effect on the suppression of austenite grain growth. The dissolution of NbC and the coarsening of TiN reduce the pinning pressure on grain boundaries, which contributes to the growth of austenite grain.

## 4. Conclusions

In this study, the effect of precipitate morphology on austenite grain growth in a Nb-Ti-Al steel was investigated. The conclusions are summarized as follows:

(1) The qualitative and quantitative analysis of precipitates by TEM observation showed that three types of precipitates, NbC, TiN, and AlN, existed in the Nb-Ti-Al steel after isothermal austenitization treatments. Both NbC and TiN precipitates significantly suppressed austenite grain growth. Because of small number, AlN precipitates hardly play any role in suppressing austenite grain growth.

(2) By calculating the pinning pressure, it is found that the dissolution and coarsening of precipitates weaken the pinning of austenite grain boundaries. With the increasing of pseudo-carburizing time, the volume fraction and pinning pressure of precipitates decreased. The total pinning pressure decreased from 14.23 MPa to 3.17 MPa at 950 °C and from 3.5 MPa to 0.85 MPa at 1050 °C. Combined with the small size of NbC and high thermal stability of TiN, the austenite grain growth can be effectively suppressed. When the holding time was 5 h, the average grain size was 14.3 μm at 950 °C and 66.2 μm at 1050 °C, and the duplex grain structure was not observed.

## Figures and Tables

**Table 1:** Chemical composition of the investigated steels (Main alloying elements).

Element	C	Si	Mn	Cr	Nb	Ti	Al	[N]
Wt.%	0.23	0.23	0.70	1.10	0.055	0.030	0.036	0.0134

## Data Availability

The data presented in this study are available on request from the corresponding author.
